# Healthcare disparities: patients’ perspectives on barriers to joint replacement

**DOI:** 10.1186/s12891-023-07096-0

**Published:** 2023-12-18

**Authors:** Susan M. Goodman, Insa Mannstadt, J. Alex B. Gibbons, Mangala Rajan, Anne Bass, Linda Russell, Bella Mehta, Mark Figgie, Michael L. Parks, Shilpa Venkatachalam, W. Benjamin Nowell, Collin Brantner, Geyanne Lui, Andrea Card, Peggy Leung, Henry Tischler, Sarah R. Young, Iris Navarro-Millán

**Affiliations:** 1 Department of Medicine, Weill Cornell Medicine, New York, NY 10021 USA; 2 Department of Rheumatology, Hospital for Special Surgery, 535 E 70th Street, New York, NY 10021 USA; 3 Department of Social Work, Binghamton University, Binghamton, NY USA; 4 Global Healthy Living Foundation Inc, Upper Nyack, NY USA; 5 Department of Medicine, Columbia University Vagelos Physician of College and Surgeons, New York, NY USA; 6 Department of Medicine, Donald and Barbara Zucker School of Medicine at Hofstra/Northwell, Hempstead, NY USA; 7 Department of Medicine, New York Institute of Technology College of Osteopathic Medicine, Glen Head, New York, NY USA; 8 Department of Orthopedic Surgery, New York-Presbyterian Brooklyn Methodist Hospital, New York, NY USA; 9 Department of Orthopedic Surgery, Hospital for Special Surgery, 535 E 70th Street, New York, NY 10021 USA

**Keywords:** Access to care, Arthroplasty, Mixed methods, Race/ethnicity, Surveys

## Abstract

**Objective:**

Racial and ethnic disparities in arthroplasty utilization are evident, but the reasons are not known. We aimed to identify concerns that may contribute to barriers to arthroplasty from the patient’s perspective.

**Methods:**

We identified patients’ concerns about arthroplasty by performing a mixed methods study. Themes identified during semi-structured interviews with Black and Hispanic patients with advanced symptomatic hip or knee arthritis were used to develop a questionnaire to quantify and prioritize their concerns. Multiple linear and logistic regression analyses were conducted to determine the association between race/ethnicity and the importance of each theme. Models were adjusted for sex, insurance, education, HOOS, JR/KOOS, JR, and discussion of joint replacement with a doctor.

**Results:**

Interviews with eight participants reached saturation and provided five themes used to develop a survey answered by 738 (24%) participants; 75.5% White, 10.3% Black, 8.7% Hispanic, 3.9% Asian/Other. Responses were significantly different between groups (*p* < 0.05). Themes identified were “Trust in the surgeon” “Recovery”, “Cost/Insurance”, “Surgical outcome”, and “Personal suitability/timing”. Compared to Whites, Blacks were two-fold, Hispanics four-fold more likely to rate “Trust in the surgeon” as very/extremely important. Blacks were almost three times and Hispanics over six times more likely to rate “Recovery” as very/extremely important.

**Conclusion:**

We identified factors of importance to patients that may contribute to barriers to arthroplasty, with marked differences between Blacks, Hispanics, and Whites.

**Supplementary Information:**

The online version contains supplementary material available at 10.1186/s12891-023-07096-0.

## Background

Racial and ethnic disparities in healthcare include persistent underutilization of total hip (THR) and total knee replacement (TKR) surgery for Blacks and Hispanics compared to Whites, and while Hispanics may have less OA, there is a higher prevalence of symptomatic and severe osteoarthritis (OA) among Blacks [1–5]. Racial disparities in arthroplasty outcomes are also well documented and include increased mortality and more revision surgery, as well as worse pain and function both before and after surgery, which may reflect delays in accessing care [6–12]. Patients have identified concerns about improvements in pain and function and surgical complications as important concerns regarding arthroplasty utilization, but participants in most studies have undergone arthroplasty, while the concerns in patients who have not undergone arthroplasty or even sought specialty care have been harder to assess [13, 14]. The patient’s perspectives and concerns about arthroplasty are not well understood, limiting the possibility of interventions for change. In addition, since Blacks are more likely to live in poverty, results linked to race may be confounded by poverty, and the utilization and outcomes of arthroplasty are similar for those from poor neighborhoods and for those without much education [10, 11, 15, 16]. Blacks, Hispanics, and individuals from low-income communities have worse pain and function at the time they undergo THR and TKR than those from wealthier communities, and since baseline status impacts outcomes, delays in care have long-term consequences [2, 11]. The reasons for delay in care by these populations are unknown, as studies have not identified the concerns from the patient’s perspective. Since THR and TKR are elective procedures, patients’ preferences are a critical component that needs to be included in a patient centered approach, to improve appropriate utilization of arthroplasty [2, 17]. Potentially important factors described include racial or cultural concordance of the provider, patient trust in medical care, as well as insurance and economic factors, but the patient’s perspective about orthopedic care has not been analyzed [7, 18–20].

The purpose of this sequential qualitative-quantitative mixed methods study was to identify concerns about arthroplasty from the patient’s perspective, then quantify and prioritize their concerns. Concerns identified in semi-structured interviews with Black and Hispanic patients with advanced symptomatic hip (HOA) and knee osteoarthritis (KOA) were used to develop a survey that we deployed to a wider population, to quantify and prioritize the concerns that may contribute to barriers to orthopedic care.

## Methods

### Design

This study employed a sequential 2-stage qualitative-quantitative research design, incorporating first a qualitative approach involving patient interviews and second a quantitative questionnaire administered to a prospective cohort. Study components were approved by the ethics committee of the Weill Cornell Institutional Review Board (WCM-IRB) [Protocol number: 1807019476]. All participants provided written informed consent and the study was undertaken in accordance with the Declaration of Helsinki.

### Qualitative component

We developed a project-working group including staff and community patient partners to develop the interview topic guide. Black and Hispanic patients with advanced knee or hip OA patients with were identified by their treating physicians for recruitment. We used purposeful sampling within the federally qualified Long Island City Community Healthcare Network (LICCHN) where 43% of the population lives below the poverty level. We scheduled multiple focus groups but because of the COVID-19 pandemic lockdown after one focus group switched to semi-structured interviews.

The inclusion criteria for the semi-structured interviews and focus group were being ≥18 years of age, Black or Hispanic, an English speaker and have limiting pain and poor function defined by osteoarthritis relevant short forms of the hip disability and osteoarthritis outcome score (HOOS, JR) and knee injury and osteoarthritis outcome score (KOOS, JR) surveys (score > 60 on a 1–100 scale, higher = worse) [21, 22]. We excluded individuals with prior THR or TKR. We collected demographic and clinical information including age, sex, comorbidities, medications, and employment status in a pre-interview questionnaire.

#### Pre-interview questionnaire

We collected responses to a short questionnaire with participants of the interviews and focus group regarding their demographic information and medical history.

#### Qualitative topic guide and qualitative data analysis

The interview topic guide was informed by the Socioecological Framework [23] to elicit perspectives of Black and Hispanic individuals with advanced osteoarthritis of the hip or knee and their perceptions of THR and TKR. We pilot tested the topic guide with two individuals with severe osteoarthritis before recruiting participants for the semi-structured interviews. The topic guide was refined following pilot testing, and the data from the two individuals who piloted it were excluded from the final analysis (Supplementary Table S2). A trained investigator (INM) conducted and supervised the focus group and all interviews for the qualitative component. The interviews and focus group were recorded, transcribed verbatim and analyzed thematically by two independent coders (SRY; INM) using NVivo software Version 12. Following the initial two interviews, the independent coders addressed coding discrepancies and proceeded to code the remaining interviews using the same codebook. Semi-structured interviews were conducted and analyzed until no new themes emerged, indicating thematic saturation. The themes and quotes that emerged during the qualitative phase were used to develop the survey questions.

### Quantitative component methods

We developed a survey informed by the qualitative data collected. The initial 30-question survey was scored on a five-level Likert scale (1 = Not at all important; 2 = A little important; 3 = Somewhat important; 4 = Very Important; 5 = Extremely important) [24]. The survey was translated to Spanish and deployed in both English and Spanish via email to patients identified at the Cornell Internal Medicine Practice and the rheumatology clinic at Hospital for Special Surgery, The Brooklyn Methodist Hospital, ArthritisPower [25], and the Global Healthy Living Foundation’s (GHLF) Spanish-language support network, CreakyJoints Español [26], between 2/27/2020 and 7/10/2022.

#### Quantitative survey statistical analysis

Descriptive statistics were employed to characterize the participants and determine the prevalence of the barriers to undergoing THR or TKR by race/ethnicity. Initial analysis profiled all 30 questions by race/ethnicity, and then calculated reliability metrics using Cronbach’s alpha for each theme. We then conducted a factor analysis to identify the dominant concerns about THR or TKR and reduce the number of factors to be listed for further analysis. We chose a final factor solution (where the eigenvalue was > 1) and subsequently rechecked the reliability of the updated factors. Finally, we calculated mean factor scores for each respondent using the variables with the highest factor loadings on an orthogonally rotated factor matrix. Our final questionnaire contained 21 questions across five themes (Table 1), with high reliability metrics (Cronbach’s alpha 0.75–0.97). Across all the 21 questions, less than 5% of respondents had missing data. Hence, all analysis has been performed on those respondents with complete information.

**Table 1:** Barriers to Arthroplasty Survey

The following statements are factors people think about when getting a joint replacement. Please check the box that shows how important these items would be if you were thinking about getting a joint replacement					
	Not at all	A little	Somewhat	Very	Extremely
**Cost and Insurance**					
Cost of a joint replacement					
Cost of the co-pay for a joint replacement					
Cost of a co-pay for physical therapy after joint replacement					
Insurance status					
**Recovery**					
Availability of someone to help me recover from a joint replacement					
Availability to take care of my family/friends while I undergo joint replacement					
Concern of being healthy enough to undergo joint replacement surgery					
Accessing transportation to get to physical therapy appointments					
Finding good physical therapy centers in my community					
Concerns about how hard the recovery after a joint replacement will be					
**Trust in the surgeon**					
Finding a surgeon I trust					
Figuring out how to find a qualified and experienced surgeon					
Finding a surgeon who understands what I need					
**Surgical outcome**					
Fear that I will need another joint replacement after the first one because I am young					
Fear that a joint replacement will not help me walk and function better					
Fear that the joint replacement will not improve my pain					
**Timing**					
Having a joint replacement is the last resort, and I think I should wait longer					
Having many medical problems and having a joint replacement is not a priority now					
Not doing everything I can do (like lose weight) to avoid having a joint replacement					
Not having bad enough joint pain to have a joint replacement					
Not having enough information to decide about having a joint replacement					

We dichotomized each concern (factor) with the top two mean responses (4 = very important; 5 = extremely important) coded as 1 (very/extremely important) and the rest coded as 0 (Not as important). Each concern (factor) was categorized by race and ethnicity. We then conducted crude and multivariable analysis with each dichotomized factor used as a dependent variable in the model to determine the association between race/ethnicity and the importance of each factor, after adjusting for sex, insurance status, education level, HOOS, JR/KOOS, JR scores and discussion of joint replacement with a doctor. Logistic regression models were chosen after running linear and modified Poisson models based on the lowest AIC (Akaike Information Criterion) score, a measure that assesses the fit of the model.

## Results

### Qualitative interviews and focus group

Thematic saturation was achieved through the analysis of interviews conducted with one focus group and six individual semi-structured interviews (*n* = 8 individuals), held between 12/1/2018 and 9/19/2019 (9.8% of screened). There were six (75%) females (three Black, two Hispanic, one Asian/Other, mean age 58.9) and two (25%) males (one Black, one White, mean age 55 years), and all had severe KOA. We identified seven initial themes that captured the prominent concerns to proceed with THR and/or TKR. Themes included: *trust in the surgeon, cost and insurance, surgical outcomes* and *improvement in pain and function after surgery*, *timing, trust in medical establishment and doctors, and recovery.* Table 2 presents the themes with corresponding quotes and constructs from the socioecological framework.

**Table 2:** Interview Themes Mapped to Socio Ecological Framework with Quotes

Concerns about Arthroplasty	Socioecological Framework Construct	Interview Quotes
**Cost and insurance**	Cost of a joint replacement. (Structures and systems)	*“That is (cost) important … Because I’m only getting Social Security money.* ***I don’t have any other income coming in. What’s the cost?****”* (P6) *“I’m reluctant to see a specialist for my knee because I’ll have to see him once or twice a month and that’s* ***a copay that I have to pay.****” (P5)*
**Recovery**	Availability of someone to help me recover, to take care of my family/friends. (Interpersonal)	*“If I have support that’s all because, to be honest,* ***I don’t have anybody at home to support me*** *right here because my children are grown. They’re all on their own. I would have to fight it all on my own.”* (P4) *“Well, if I get a knee replacement,* ***I’m definitely going to need assistance, because I’ve got a handicapped daughter*** *in my apartment. She is 50 years old. She’s having the problem walking because she has cerebral palsy.”* (P1)
	Finding good physical therapy centers in my community (Community or Institutions and Organizations)	“***In my neighborhood there’s not really any places that are comprehensive*** *in terms of, in terms of physical therapy or anything like that. They’re not really sophisticated or well-well-built places for physical therapy.”* (FG1)
**Trust in surgeon**	Finding a surgeon I trust. (Individual and Interpersonal)	“***I would know how good the surgeon is****. I would like to know some results of how many patients he had done things for and they’re still walking,* ***how good a job he did with them, and does he still keep in contact with his patients****.”* (P1)
	Figuring out how to find a qualified and experienced surgeon (Individual, Interpersonal, Institutional and Organization)	*“* ***I would want an experienced surgeon****. I would like to know about how many surgeries he has done, how long he’s been in that field, the hospital that he works at.* *How many successful rates are there with him?”* (P3)
**Surgical outcome**	Fear that a joint replacement will not help me walk and function better, will not improve my pain. (Individual)	*“* ***My outcome (from surgery) would have to be very important*** *because I have to be sure that I’m going to be fine. It won’t be taking too long.* ***I won’t be staying home too long.****”* (P4) *“* ***If my walking will be improved*** *(walking pain-free)”. (P6)*
**Timing**	Having many medical problems and having a joint replacement is not a priority now. (Individual)	“I’ve been working on [deciding whether to have the surgery], but because **there’s been other health issues that have been coming up with me, we’re trying to do one thing at a time**.” (P2)
	Not doing everything I can (like lose weight) to avoid having a joint replacement.	*“* ***I need to work on my diet even more. I need to work on my little exercise, just my whole lifestyle needs to change.*** *My weight plays a bit part in it, believe it or not. I know it does.”* (P3)
**Mistrust**	Having someone I know have a bad result from a joint replacement. (Interpersonal, Institutions and Organizations, Community)	*“* ***After you see somebody that took the surgery and they’re not happy with it or anything like that, that sort of scare me.*** *I wouldn’t go into the surgery right now. No.”* (P6)
	Not having any trust in doctors or hospitals. (Individual, Interpersonal, Organization)	***“Some of them (doctors) are in it for the money*** *and some of them are in it because they genuinely care about their patients. Some are just not good at being doctors. Sometimes you get those too.” (FG1)* *“They didn’t really care, as long as I came to the appointment* ***so that they could get paid or whatever the case may be****. And you could tell that that’s what their main interest was, you know? But it’s because I’ve seen doctors, I’ve just seen doctors.”* (FG1)

Participant1,2,3,4,5,6- Patient numbers. FG1- Focus Group 1

### Survey results

Between February 27, 2020, and July 10, 2022, 738 (24% response) participants returned surveys, primarily from HSS (Table 3). The majority were females and 19% of the participants were either Black or Hispanic. Due to the limited size and heterogeneity of the “Asian/Other” group, we refrain from further discussing this group although they were not excluded from the analysis. Average HOOS, JR score was 58.9 and KOOS, JR score was 51.7, indicating moderate to severe symptoms, with no difference in reported hip or knee pain between groups.

**Table 3:** Characteristics of Survey Responders Grouped by Race/Ethnicity^a^

	Overall	Race Category			Significance Test*
	Cohort *N* = 738	Black (B) *N* = 76	White (W) *N* = 556	Hispanic (H) *N* = 64	*p*-value
Sociodemographic variables					
Age, years (mean, SD)	59.3 (10.8)	59.9 (13.4)	59.8 (10.2)	53.7 (9.8)	< 0.01
Female (%)	88.0	90.8	87.9	87.3	
Education level (%)					
Some high school	8.9	14.5	5.9	20.3	< 0.01
Some college	37.7	51.3	35.3	34.4	
College graduate	53.5	34.2	58.0	45.3	
Insurance status^b^ (%)					
Medicare	46.2	50.0	48.8	32.8	< 0.01
Medicaid	18.2	27.6	16.9	20.3	
Private	49.3	40.8	51.4	53.1	
Uninsured	4.9	7.9	3.4	10.9	
Pain and disability attributed to arthritis (mean, SD)					
Pain in hip/knee (Y/N) (%)	96.9	93.4	97.5	93.8	< 0.1
Pain VAS (0–100) (0–100)	61.5 (23.0)	65.7 (28.5)	60.5 (21.9)	60.6 (24.7)	< 0.05
HOOS, JR (0–24)	58.9 (23.9)	61.3 (28.1)	59.6 (20.0)	55.2 (23.9)	< 0.1
KOOS, JR (0–26)	51.7 (20.3)	50.3 (25.3)	52.7 (19.8)	49.0 (18.8)	< 0.05
Providers seen to evaluate arthritis (%)					
Primary care physician	48.0	39.5	51.8	42.2	< 0.01
Orthopedist	47.6	56.6	48.9	35.9	< 0.05
Rheumatologist	57.7	31.6	60.6	67.2	< 0.01
No one	4.6	5.3	4.7	6.3	
Other	8.3	10.5	7.9	7.8	
Discussed arthroplasty with provider	51.4	65.8	52.5	29.7	< 0.01
Treatments tried for arthritis (%)					
Over the counter medication	81.8	72.4	85.6	70.3	< 0.01
Physical therapy	61.8	61.8	63.5	53.1	
Acupuncture	15.0	10.5	15.7	14.1	
Braces	24.5	23.7	25.5	20.3	
Joint injection	59.8	51.3	63.5	43.75	< 0.01
Topical creams/salves	64.9	56.6	67.3	67.2	
Prescription medication	75.9	65.8	77.2	81.3	< 0.1
Other	10.7	4.0	11.3	14.1	
None	2.6	5.3	2.3	3.1	

*Significance tests are Chi-square/Fisher’s exact tests comparing all race categories (Black, White, Hispanic)

^a^Unless otherwise stated, all values presented represent the percentage of participants per variable

^b^Patients can be on multiple insurance categories

A greater percentage of Blacks (57%) had visited an orthopedist compared to Hispanics (36%) or Whites (49%). Compared to Blacks and Hispanics, a larger percentage of White participants attempted to alleviate their symptoms through treatments such as over-the-counter pain medications, prescription medications, and joint injections.

### Factor analysis

**Fig. 1:**
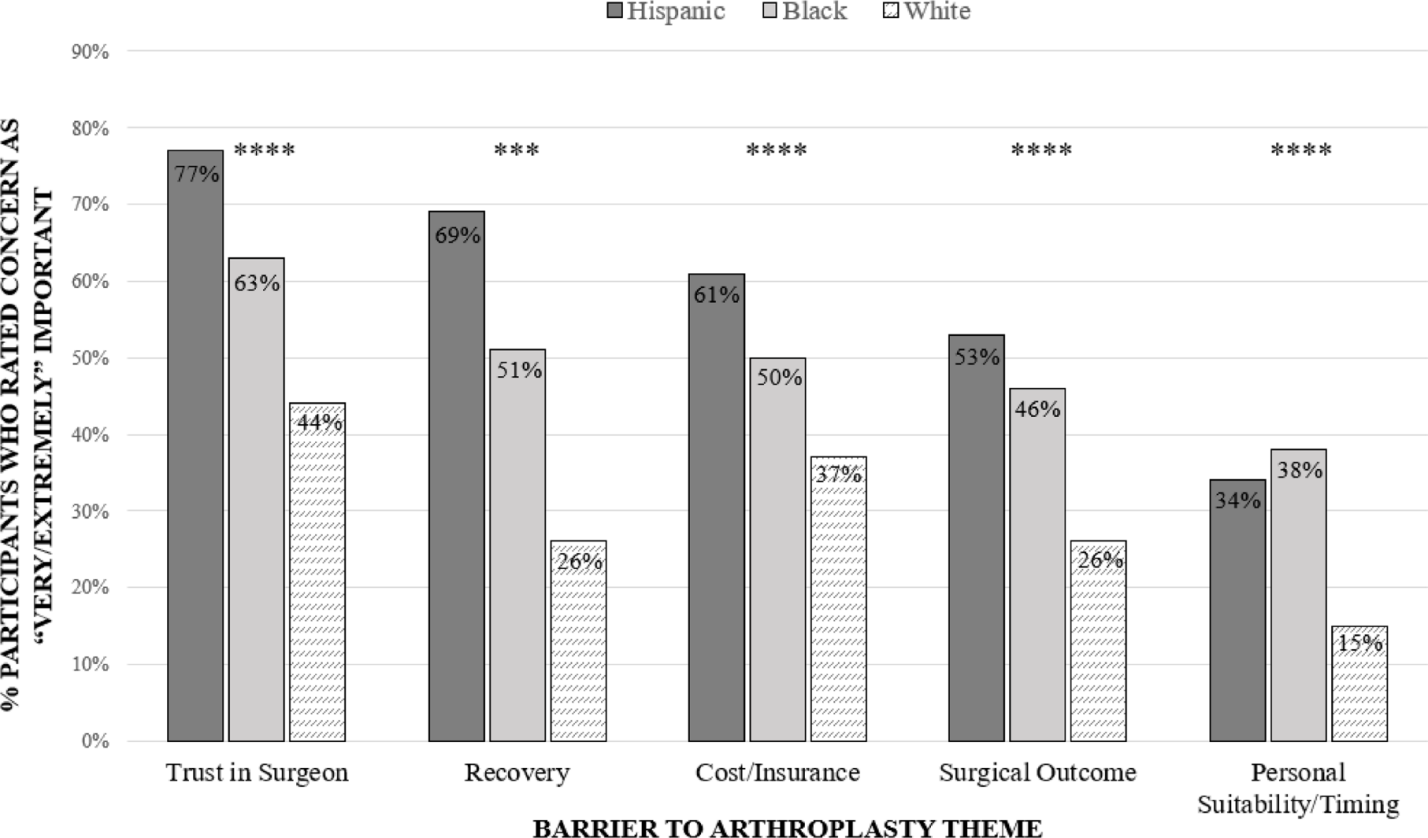
Racial and Ethnic differences in proportion of Very/Extremely important ratings to the identified concerns to arthroplasty. Statistical significance markers: p < 0.1; * p < 0.05; ** p < 0.01; *** p < 0.001; ****p < 0.0001

The factor analysis resulted in five dominant themes identified as concerns for joint replacement: *1. Trust in the surgeon, 2. Recovery, 3. Cost and insurance, 4. Surgical outcome* and *5. Timing.* (Supplementary Table S1) Dichotomized scores *very/extremely important* vs. *Not as important* varied significantly by race and ethnicity, with *p*-values < 0.01 for all comparisons (Fig. 1). 63.2% of Blacks and 77.8% of Hispanics rated “*Trust in the surgeon”* as very/extremely important compared to 43.6% of Whites. “*Recovery”* was very/extremely important for 51.3% of Blacks, 69.8% of Hispanics, and 26.4% of Whites. “*Cost and insurance”* were very/extremely important for 59.3% of Blacks, 37.7% of Whites, and 61.9% of Hispanics. “*Surgical outcome”* was very/extremely important to 46.7% of Blacks, 26.6% of Whites, and 54.0% of Hispanics. “*Timing”* was rated very/extremely important by 36.5% of Blacks compared to 15.6% of White respondents.

After adjusting for sex, insurance status, education level, HOOS, JR/KOOS, JR scores and whether they have discussed the option of joint replacement with a doctor (Table 4), Blacks were two-fold more likely to consider *“Trust in the surgeon”* as very/extremely important compared to Whites (Adjusted Odds Ratio (aOR) 2.20, 95% CI 1.31, 4.70). Hispanics were more than four-fold more likely to rate *“Trust in the surgeon”* as very/extremely important compared to Whites (aOR 4.27, 95%CI 2.22,8.20). Blacks (aOR 2.85, 95% CI 1.67,4.86) had almost three-fold higher likelihood than Whites and Hispanics (aOR 6.52, 95% CI 3.49,12.18) had a six-fold greater likelihood than Whites of rating *Recovery* as *very/extremely important*. Blacks (aOR 2.27, 95% CI 1.33, 3.85) and Hispanics (aOR 2.73, 95% CI 1.54, 4.85) were twice as likely to rate *“Surgical outcome*” as very/extremely important compared to Whites. Blacks (aOR 3.92, 95% CI 2.16,7.13) were almost four times and Hispanics (aOR 2.20, 95% CI 1.19, 4.09) more than two-fold more likely to rate the *“Timing”* of the procedure as very/extremely important compared to Whites. “*Cost and insurance”* were similar between all racial and ethnic groups (Table 4).

**Table 4:** Multivariable Logistic Regression Analysis Associating Race/Ethnicity with Barriers to Arthroplasty

Barriers for joint replacement theme	Race/Ethnicity	Crude Odds Ratio (95 C.I.)	Adjusted Odds Ratio (95 C.I.)^a^
Trust in the surgeon	Black	**2.21 (1.35, 3.64)**	**2.20 (1.31, 4.70)**
	Hispanic	**4.52 (2.44, 8.38)**	**4.27 (2.22, 8.20)**
	Asian/Other	**2.94 (1.42, 6.09)**	**2.52 (1.11, 5.72)**
	White	*Reference*	*Reference*
Recovery	Black	**2.94 (1.80, 4.79)**	**2.85 (1.67, 4.86)**
	Hispanic	**6.45 (3.65, 11.42)**	**6.52 (3.49, 12.18)**
	Asian/Other	**2.12 (1.08, 4.18)**	1.77 (0.81, 3.85)
	White	*Reference*	*Reference*
Cost and insurance	Black	1.61 (0.99, 2.61)	1.54 (0.90, 2.65)
	Hispanic	**2.69 (1.57, 4.60)**	1.73 (0.95, 3.14)
	Asian/Other	1.57 (0.80, 3.05)	1.10 (0.49, 2.45)
	White	*Reference*	*Reference*
Surgical outcome	Black	**2.42 (1.48, 3.96)**	**2.27 (1.33, 3.85)**
	Hispanic	**3.24 (1.91, 5.41)**	**2.73 (1.54, 4.85)**
	Asian/Other	1.33 (0.65, 2.71)	1.10 (0.49, 2.46)
	White	*Reference*	*Reference*
Timing	Black	**3.12 (1.84, 5.28)**	**3.92 (2.16, 7.13)**
	Hispanic	**2.91 (1.65, 5.13)**	**2.20 (1.19, 4.09)**
	Asian/Other	1.31 (0.56, 3.09)	1.18 (0.44, 2.74)
	White	*Reference*	*Reference*

(^a^) Models adjusted for sex, education level, HOOS, JR KOOS, JR Score, Insurance Status, and discussion of knee/hip surgery with a doctor

## Discussion

Trust in the surgeon, Recovery, Cost and insurance, Surgical outcome, and Timing for having knee replacement were the most important concerns with joint replacement among underrepresented minority groups of Blacks and Hispanics. Whites, Blacks and Hispanics also placed different values on each of these concerns. Hispanics had the highest likelihood of assigning “*very/extremely important*” to *Trust in the surgeon*, *Recovery*, *Cost and insurance,* and *Surgical outcome* compared to any other racial and ethnic group. The prevalence of these concerns rated *very/extremely important* ranged between 15 and 37% among White patients with *Trust in the surgeon* at 47% compared to 63.2% of Blacks and 77.8% of Hispanics. This emphasizes that the concerns regarding the use of THR or TKR vary greatly among racial and ethnic groups and the importance of addressing the concerns identified in this study to promote the use of THR and TKR among minority patients.

While many studies have benchmarked the persistent racial and ethnic disparities in arthroplasty utilization and outcomes linked to social factors such as social deprivation or race [3, 5, 27], there is little information about the concerns regarding arthroplasty utilization from the patients’ perspective. Furthermore, the duration between orthopedic referral and surgery is similar for White, Black, and Hispanic patients. However, Black and Hispanic patients present for arthroplasty later than White patients, with more severe preoperative pain and functional limitations, suggesting that different concerns between the groups studied may contribute to barriers to arthroplasty arising at multiple points throughout the care process [11, 28].

In this study, we found that a significantly higher proportion of Blacks and Hispanics than Whites rated *Trust in the surgeon* as a very/extremely important concern. While almost half of the patients in this study have seen an orthopedist, we have no data on physician race concordance. Race concordance improves trust and communication, and patients are more likely to participate in decision making when the physician is the same race [29, 30]. Given that Blacks represent only 6% of physicians overall and < 2% of the orthopedic surgeons, and Hispanics represent 18% of the population and 5% of physicians, it is unlikely that the Black and Hispanic patients were seen by Black or Hispanic orthopedists [31]. In a study of over 130,000 patients in the Kaiser system, only 10% of Blacks and 11% of Hispanics had the same race/ethnicity as their physicians, and only 24% of Spanish-speaking patients were linguistically concordant [32]. Although race and language concordance were not elicited as a concern during our interviews, it is possible that the concerns expressed by Black and Hispanic patients reflects prior experiences with race, language, and ethnicity discordant physicians.

It is also possible that minority groups know only about the resources available to them in their communities, like physical therapy (PT) centers. During the interviews, participants expressed that they had reservations about the quality of the medical facilities available in their community but were unsure where to find better options they could rely on. Blacks and Hispanics were markedly more likely to consider *Recovery,* a theme that encompasses post-operative physical therapy, including access to PT which is important for optimal arthroplasty outcomes, to be very/extremely important compared to Whites. Prior work using administrative data on almost 24,000 patients has demonstrated that Blacks and Hispanics are less likely to receive PT after TKR than Whites [33]. In a study holding focus groups 3 months after arthroplasty, Black and White participants described differences regarding barriers to PT that included economic factors such as co-pays and time off work, as well as difficulty finding transportation to PT sessions [34]. The basis for the disparity in utilization and access to PT during arthroplasty recovery is not known but may be contributing to the concerns about arthroplasty recovery described in our study. This highlights a less apparent potential barrier to arthroplasty, which is the difficulty in navigating the healthcare system and is reflected in the concerns expressed about surgical recovery.

Blacks and Hispanics endorsed concerns about caring for their family during their recovery as well as concerns about the availability of others to care for them. A study of long term unpaid caregivers who provided substantial help with healthcare found that caretakers are five times less likely to participate in personal activities as well as three times more likely to report a loss in work productivity compared to those not providing help [35]. As our cohort is demographically alike, similar concerns might be applicable.

Black and Hispanic participants were significantly more likely to rate *very/extremely high importance* to *Surgical outcome* and improving their pain and function than Whites. These concerns may accurately reflect the frequent use of hospitals with low arthroplasty volume, where many minority groups receive care that are associated with worse scores for pain and function, more frequent postoperative complications including emergency room visits for Blacks and Hispanics, and greater risk of revision surgery reported for Black patients [9, 36–39]. During the interviews, patients expressed concerns about joint replacement procedures, especially after learning about someone who experienced a negative outcome. They expressed uncertainty about identifying a suitable facility for joint replacement, aside from the one where a friend or a person known to them had a negative experience. Moreover, if they were aware of another option, they raised concerns about whether their insurance would cover the cost.

Most participants in all groups considered *Timing* of surgery related to medical optimization or symptom severity *“not as important*”, although twice as many Blacks and Hispanics reported “*very/extremely important*” concerns about medical problems, weight reduction, or “bad enough” pain compared to Whites. This concern may relate to disparities among Blacks and Hispanics regarding diabetes control, hypertension control, and the high prevalence of obesity and smoking which may preclude surgical clearance [40, 41]. Therefore, the barriers for utilization of joint replacement are related but not limited to the procedure for joint replacement but also to the disparities that exist across medical care for chronic conditions. Despite the recommendation of the American College of Rheumatology/American Association of Hip and Knee Surgeon Guideline advising against strict cutpoints for medical conditions [42], poor control of chronic conditions may make surgeons less likely to offer these procedures to these patients until they achieve better glycemic control or better weight [43, 44], while many of them lack the access or best resources to achieve those goals.

While a majority of Hispanics considered *Cost and insurance* to be a “*very/extremely important*” factor, only half of Black participants and fewer White participants considered *cost and insurance* to be “*very/extremely important*”. The association of higher levels of social deprivation or Medicaid insurance with lower levels of arthroplasty utilization is well described [45, 46] but an experimental hospital reimbursement model aimed to increase access to TKR did not increase TKR utilization among low-income patients [47]. The importance of *Cost and insurance* from the patient’s perspective includes concerns about co-pays for both the procedure as well as factors during recovery like PT.

One strength of this study is our mixed methods approach. We developed the survey using data obtained through carefully analyzed interviews among underrepresented minority groups living in an impoverished community. The survey was then widely distributed and enabled us to quantify and prioritize the identified barriers in a larger population across multiple sites and multiple states across the USA, improving the generalizability of our results.

A limitation of this study was due to the COVID-19 pandemic, which prevented use of focus groups as originally planned and led to the use of semi-structured phone interviews. Despite this limitation, thematic saturation was achieved. We also faced challenges in obtaining questionnaire responses from Black and Hispanic individuals. To address this, the study reached out to Creaky Joints Español, a Spanish language support group, and Brooklyn Methodist Hospital, a predominantly Black community practice, which increased the non-white response to almost 25% of participants. The consistency of views on the barriers to arthroplasty between the qualitative phase and survey phase in the Black and Hispanic participants, despite representing 25% of responses, indicates that the survey did capture the major concerns about arthroplasty for the Black and Hispanic population. The survey response rate was low overall, as would be expected in our target population. However, since we have no information on non-respondents, it is not possible to determine if there were meaningful differences between groups. Additionally, our Hispanic cohort was largely recruited from the highly engaged *Creaky Joints* Spanish Language Support Group, which may have influenced our results. As we did not require a validated diagnosis of arthritis in survey participants, we may have included those who would not have a condition treatable by joint replacement. However, we wanted to include those who thought they had symptomatic arthritis of the hip or knee to understand their concerns about arthroplasty, including potential delays in initial consultation for diagnosis.

## Conclusion

In summary, we have identified concerns about arthroplasty from the patients’ perspective and report that there are significant differences between Blacks, Hispanics, and Whites in the factors that are most important to them. Solutions to joint replacement utilization disparities will require addressing those concerns that emerged from this study that may contribute to barriers to care, such as access to qualified physicians. Additionally, addressing health disparities related to the treatment of chronic conditions, which were identified as barriers for the utilization of joint replacement in the Black and Hispanic population, is also necessary.

### Supplementary Information

**Additional file 1.**

**Additional file 2.**

## Data Availability

The datasets used and/or analyzed during the current study are available from the corresponding author on reasonable request.

## Abbreviations

GHLF: Global healthy living foundation
THR: Total hip replacement
TKR: Total knee replacement
OA: Osteoarthritis
HOA: Hip osteoarthritis
KOA: Knee osteoarthritis
HOOS, JR: Short form of hip disability and osteoarthritis outcome score
KOOS, JR: Short form of knee injury and osteoarthritis outcome score
LICCHN: Long island city community healthcare network
AIC: Akaike information criterion
aOR: Adjusted odds ratio
PT: Physical therapy
WCM-IRB: Weill cornell institutional review board
