# Supplementary material for: Healthcare disparities: patients’ perspectives on barriers to joint replacement
Source: BMC Musculoskelet Disord. 2023 Dec 18;24:976. doi: 10.1186/s12891-023-07096-0 (PMC10726517; doi:10.1186/s12891-023-07096-0)
Supplement: Supplementary file 1 — Additional file 1. [file 12891_2023_7096_MOESM1_ESM.docx]

**Supplementary Table S1:** Distribution of Rated Importance (% Rated Very/Extremely Important) Per Factor and of Survey Items within Each Factor: Overall and Stratified by Race

| **Survey Items Within Theme (Cronbach α)** | **Overall** | **Race Stratification** | | | **Significance test (P-value)** |
| --- | --- | --- | --- | --- | --- |
|  | **Cohort**  **N=738** | **Black (B)**  **N = 76** | **White (W)**  **N = 556** | **Hispanic (H)**  **N=64** |  |
| **1. Trust in surgeon (α=0.97)** | **49.9** | **63.2** | **43.6** | **77.8** | **<0.01** |
| Finding a surgeon I trust | 51.2 | 63.2 | 45.5 | 79.7 |  |
| Finding a surgeon who understands what I need | 52.4 | 60.5 | 47.5 | 75 |  |
| Figuring out how to find a qualified, experienced surgeon | 44.8 | 61.8 | 38.3 | 75 |  |
| **2. Recovery (α=0.86)** | **33.7** | **51.3** | **26.4** | **69.8** | **<0.01** |
| Availability of someone to help me recover from arthroplasty | 48 | 59.2 | 43.7 | 68.8 |  |
| Availability to care for family/friends while I undergo arthroplasty | 36.4 | 42.1 | 32.2 | 64.1 |  |
| Accessing transportation to get to physical therapy appointments | 31.9 | 51.3 | 26.8 | 51.6 |  |
| Concern of being healthy enough to undergo arthroplasty | 32.1 | 46.1 | 26.6 | 59.4 |  |
| Finding good physical therapy centers in my community | 27.3 | 46.1 | 20.5 | 62.5 |  |
| Concerns about how hard the recovery after arthroplasty will be | 52.7 | 68.4 | 47.5 | 71.9 |  |
| **3. Cost and Insurance (α=0.90)** | **41.5** | **59.3** | **37.7** | **61.9** | **<0.01** |
| Cost of the co-pay for a joint replacement | 43.2 | 50 | 40.3 | 56.2 |  |
| Cost of a joint replacement | 38.8 | 46.1 | 34.7 | 57.8 |  |
| Cost of a co-pay for physical therapy after arthroplasty | 41.2 | 48.7 | 39 | 51.6 |  |
| Insurance status | 43.2 | 59.2 | 38.5 | 67.2 |  |
| **4. Surgical outcome (α=0.81)** | **31.3** | **46.7** | **26.6** | **54.0** | **<0.01** |
| Fear that arthroplasty will not help me walk and function better | 37.5 | 48.7 | 32 | 67.2 |  |
| Fear that arthroplasty will not improve my pain | 44.6 | 55.3 | 39.6 | 68.8 |  |
| Fear that I will need another arthroplasty after the first one because I am young | 23 | 31.6 | 20 | 42.2 |  |
| **5. Timing (α=0.75)** | **19.6** | **36.5** | **15.6** | **34.9** | **<0.01** |
| Not having bad enough joint pain to have arthroplasty | 26.6 | 40.8 | 22.7 | 42.2 |  |
| Having arthroplasty is last resort, and I think I should wait longer | 45.7 | 50 | 44.6 | 50 |  |
| Having many medical problems, having arthroplasty is not priority | 27 | 42.1 | 24.3 | 26.6 |  |
| Not doing everything I can (like lose weight) to avoid arthroplasty | 28.7 | 43.4 | 24.6 | 42.2 |  |
| Not having enough information to decide about arthroplasty | 21.8 | 43.4 | 15.5 | 46.9 |  |

*† Unless otherwise stated, all values presented represent the percentage of participants per variable. Differences between continuous variables and race/ethnicity were tested using the Wilcoxon rank sum test for 2 samples.*

*††* p<0.05; ** p<0.01; *** p<0.001; ****p<0.0001*
