# Supplementary material for: Healthcare disparities: patients’ perspectives on barriers to joint replacement
Source: BMC Musculoskelet Disord. 2023 Dec 18;24:976. doi: 10.1186/s12891-023-07096-0 (PMC10726517; doi:10.1186/s12891-023-07096-0)
Supplement: Supplementary file 2 — Additional file 2. [file 12891_2023_7096_MOESM2_ESM.docx]

**Supplementary Table S2**: Topic guide used for patient interviews

| **Q.1 Picture yourself at the moment at the moment that you realized that the pain in your knee or hip was not going away. You may have had pain before that came and went, but this episode persisted. How did this make you feel?**   - Fear? Why? - Pain? - Anxious? - Uncertain? - Other? |
| --- |
| **Q.2. What are your thoughts about undergoing joint replacement to alleviate your hip or knee pain?**   - Why do you feel that way? - Do you think that you might be a candidate for joint replacement? Why and why not? Is this the reason that you are delaying surgery? |
| **Q.3. What kind of things would you like to know about knee or hip replacement before deciding to have this procedure?**   - Do you want to know how experienced the surgeon is? Number of complications with that surgeon? Other? - Do you have a friend or relative who had surgery with a particular surgeon? Hospital? - What would you like to know about the surgeon? Hospital? - How did they, the friend that has had the surgery before, do? (The outcome of a friend or a relative regardless of who the surgeon or hospital was) - The outcome of a friend or a relative with a specific surgeon or a specific hospital? - Complications? Which are the most concerning to you and that you would like to learn in detail so that you can decide about having joint replacement? - Time to recover? - Time spent in rehabilitation. Why? - When can you go back to work after surgery? |
| **Q.4. What challenges do you anticipate after undergoing a joint replacement if a doctor recommends it to treat the severe knee or hip pain that you have from arthritis?** Why?   - What are your concerns about this procedure? The preparation? The recovery? Why? - What kind of support do you have at home that could assist with the recovery from a joint replacement surgery? - Are you concerned about things that may occur unexpectedly? - What kind of unexpected things are you concern? Which of these are the three most important? - Are you afraid that your family responsibilities that would be disrupted? What kind of responsibilities? In which way do you think that joint replacement will disrupt these responsibilities? - Would a bad experience with joint replacement by a friend or a relative influence your decision in having this procedure despite having severe pain? Why and why not? |
| **Q.5. What would make you feel motivated to have a joint replacement if a doctor recommends it to treat the severe knee or hip pain that you have from arthritis?**   - Why? - The surgeon doing the surgery? The hospital reputation? - The type of support that you have for recovery at home? |
| **Q.6. What kind of help would you have at home to assist in recovering from joint replacement? By whom?**   - Could you describe your current family or social support? Would they be available to help you after a joint replacement? In which way? |
| **Q.7. How capable do you think you are to engage in physical therapy after having knee or hip replacement? Why and why not?** |
| **Q.8. How is your trust in the health system? Why?**   - How is your trust in the health system influencing your decision of having a joint replacement? Why? - What things have influenced your trust in the health system? Why? - The experience of a friend with joint replacement? - The experience of a relative with joint replacement? - The opinion of relatives about joint replacement? - The opinion of friends about joint replacement? |
